# mTORC1/2 inhibitor and curcumin induce apoptosis through lysosomal membrane permeabilization-mediated autophagy

**DOI:** 10.1038/s41388-018-0345-6

**Published:** 2018-05-30

**Authors:** Seung Un Seo, Seon Min Woo, Hyun-Shik Lee, Sang Hyun Kim, Kyoung-jin Min, Taeg Kyu Kwon

**Affiliations:** 1 Department of Immunology, School of Medicine, Keimyung University, 2800 Dalgubeoldaero, Dalseo-Gu, Daegu, 704-701 South Korea; 2 KNU-Center for Nonlinear Dynamics, School of Life Sciences, BK21 Plus KNU Creative BioResearch Group, College of Natural Sciences, Kyungpook National University, Daegu, 41566 South Korea; 3 Department of Pharmacology, School of Medicine, Kyungpook National University, Daegu, 41944 South Korea

## Abstract

mTOR is an important regulator of cell growth and forms two complexes, mTORC1/2. In cancer, mTOR signaling is highly activated, and the regulation of this signaling, as an anti-cancer strategy, has been emphasized. However, PP242 (inhibitor of mTORC1 and mTORC2) alone did not induce human renal carcinoma cell death. In this study, we found that PP242 alone did not alter cell viability, but combined curcumin and PP242 treatment induced cell death. Combined PP242 and curcumin treatment induced Bax activation and decreased expression of Mcl-1 and Bcl-2. Furthermore, co-treatment with PP242 and curcumin-induced the downregulation of the Rictor (an mTORC2 complex protein) and Akt protein levels, and ectopic overexpression of Rictor or Akt inhibited PP242 plus curcumin induced cell death. Downregulation of Rictor increased cytosolic Ca^2+^ release from endoplasmic reticulum, which led to lysosomal damage in PP242 plus curcumin-treated cells. Furthermore, damaged lysosomes induced autophagy. Autophagy inhibitors markedly inhibited cell death. Finally, combined curcumin and PP242 treatment reduced tumor growth and induced cell death in xenograft models. Altogether, our results reveal that combined PP242 and curcumin treatment could induce autophagy-mediated cell death by reducing the expression of Rictor and Akt in renal carcinoma cells.

## Introduction

mTOR has been known as a regulator of cell growth, proliferation, metastasis, lipogenesis, and transcription. mTOR is involved in two distinct multi-protein complexes, mTORC1/2. mTORC1 contains mTOR, Raptor, GβL, and DEPTOR and phosphorylates S6K and 4EBP1. In contrast, mTORC2 contains mTOR, GβL, Rictor, Sin1, PRR5/PRR5L, and DEPTOR and regulates Akt and PKC phosphorylation and actin cytoskeleton formation [1]. Since mTOR signaling is activated in multiple types of cancers, targeting mTOR signaling is a therapeutic strategy to treat cancer. The approved everolimus and temsirolimus as rapamycin analogs have been evaluated for cancer treatment [2, 3]. However, rapamycin analogs only inhibit mTORC1, and long-term treatment with the rapamycin analog induces PI3K and Akt activation [4]. Since mTORC1 inhibits PI3K activation via the inhibitory phosphorylation of IRS-1, the chronic inhibition of mTORC1 impedes the negative feedback loop [4]. Therefore, novel inhibitors of mTORC1/2 (PP242, Torin, KU63794, and AZD8055) have been developed. However, PP242 and KU63794-induced ERK activation [5, 6], and PP242 transiently inhibits mTOR signaling in some cancer cells [6]. Therefore, identifying chemical reagents to improve the effect of mTORC1/2 inhibitors may enhance efficiency for cancer therapy.

Curcumin is a polyphenolic phytochemical compound, and it has multiple anti-cancer effects. For example, curcumin promotes apoptosis in several types of cancer cells [7–10] and inhibits migration [11, 12] and angiogenesis [13]. Furthermore, curcumin enhances the cell death of cancer cells by anti-cancer drugs treatment, including TRAIL [14–16], 5-fluorouracil and gemcitabine [17, 18]. In addition, curcumin induces non-apoptotic cell death. Curcumin-induced cell death occurs independently of caspase-3 activation in esophageal cancer cells [19] and curcumin inhibits Akt and ERK1/2 signaling pathways, leading to autophagic cell death in glioma [20]. Since such effects of curcumin on cell death depend on the concentration and specificity of cell types, further studies are urgently needed to elucidate the functions of curcumin on cancer biology.

Our results showed that curcumin enhances mTORC1/2 inhibitor-induced apoptosis and identified the molecular mechanisms by which combined PP242 and curcumin treatment induced apoptosis in human renal carcinoma cells.

## Results

### PP242 alone does not induce apoptosis in Caki cells

**Fig. 1:**
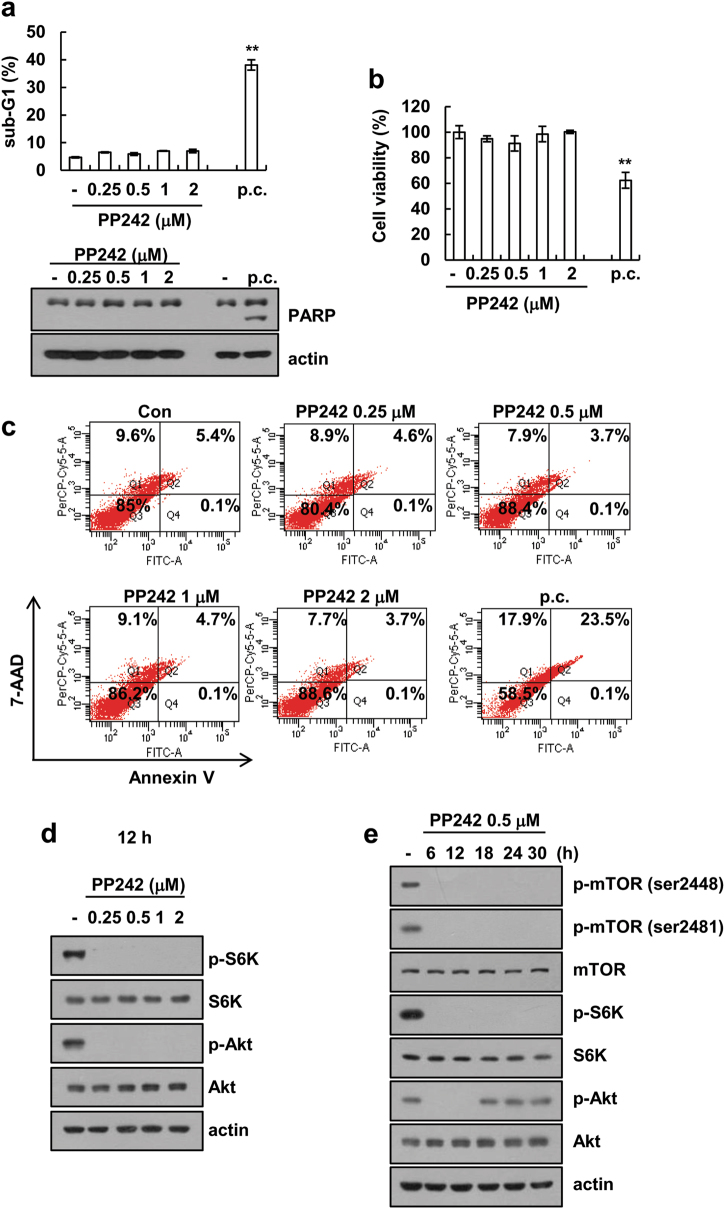
The effects of PP242 on cell death in human renal carcinoma Caki cells. **a**–**c** Caki cells were treated with 0.25–2 μM PP242 for 36 h. p.c. positive control (10 ng/ml TNF-α and 5 μg/ml cycloheximide). The level of apoptosis was assessed by measuring the sub-G1 fraction using flow cytometry in our study. Immunoblot analysis of PARP and actin (**a**). Cell viability was analyzed using XTT assay (**b**). Cell death was determined by stained with 7-AAD and Annexin V (**c**). **d** Caki cells were treated with PP242 for 12 h. Immunoblot analysis of phospho (p)-S6K, S6K, p-Akt, Akt and actin. **e** Caki cells were treated with 0.5 μM PP242 for 6–30 h. Immunoblot analysis of p-mTOR (serine 2448 and serine 2481), mTOR, p-S6K, S6K, p-Akt, Akt, and actin. The data represented in **a** and **b** are the means ± s.d. from three independent samples. **p *<* 0.01 compared to the control

Since mTORC1/2 signaling plays a pivotal role in cell survival and inhibitors of mTORC1/2 are considered anti-cancer therapeutic agents [21], we elucidated the effects of mTORC1/2 inhibitor on cell death. Combined TNF-α and cycloheximide treatment induced cell death and increased 7-AAD and Annexin V double positive cells, but PP242 (0.25–2 μM) did not induce cell death (Fig. 1a-c). Therefore, we analyzed the inhibitory effect of PP242 on mTORC1/2 signaling pathways. Because mTORC1 and mTORC2 phosphorylates Ser residues 235 and 236 of S6K and Ser residue 473 of Akt, respectively [22–24], we examined the phosphorylation of S6K and Akt to determine whether mTORC1 and mTORC2 are activated. PP242 markedly inhibited the phosphorylation of S6K and Akt, which are downstream signaling factors of mTORC1 and mTORC2 (Fig. 1d), and PP242 inhibited the phosphorylation of mTOR, Akt, and S6K within 6 h and maintained this effect for 30 h (Fig. 1e). However, decreased phosphorylation of Akt was recovered after 18 h (Fig. 1e). These results indicated that although PP242 inhibits mTORC1/2 activity, this inhibitor alone does not induce apoptosis.

### Curcumin enhances PP242-induced apoptosis

**Fig. 2:**
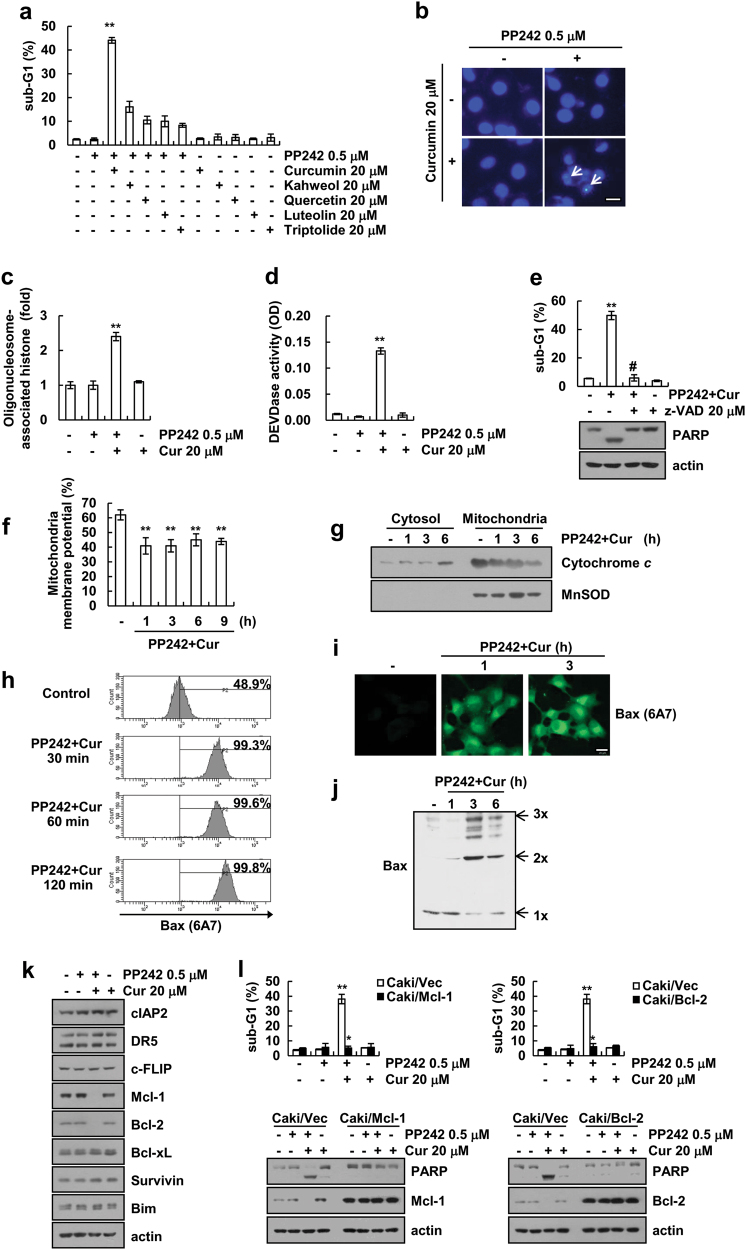
Curcumin enhances PP242-induced apoptosis. **a** Caki cells were treated with 20 μM of curcumin, kahweol, quercetin, luteolin, and triptolide in the absence or presence of 0.5 μM PP242 for 30 h. **b**–**d** Caki cells were treated with 20 μM curcumin in the absence or presence of 0.5 μM PP242 for 30 h. The nuclei were detected by DAPI staining. Scale bar = 20 μm (**b**). The cytoplasmic histone-associated DNA fragments were measured (**c**). Caspase activities were calculated using caspase-3 (DEVDase) assay kits (**d**). **e** Caki cells were treated with 20 μM curcumin plus 0.5 μM PP242 for 30 h in the presence or absence of 20 μM z-VAD-fmk (z-VAD). Immunoblot analysis of PARP and actin. **f**–**j** Caki cells were treated with 20 μM curcumin plus 0.5 μM PP242 for the indicated time periods. The mitochondrial membrane potential was measured using rhodamine123 fluorescent dye by flow cytometer (**f**). Cytosolic fractions were analyzed for cytochrome *c* release. The level of MnSOD was used as a mitochondria loading control (**g**). Cells stained for active Bax using conformation-specific antibodies. Flow cytometry (**h**) and fluorescence microscopy (**i**) detect fluorescence intensity. Scale bar = 20 μm (**i**). For Bax oligomerization assay, monomers and oligomers of Bax were quantified by western blotting (**j**). **k** Caki cells were treated with 20 μM curcumin in the presence or absence of 0.5 μM PP242 for 30 h. Immunoblot analysis of cIAP2, DR5, c-FLIP, Mcl-1, Bcl-2, Bcl-xL, Survivin, Bim, and actin. **l** Vector-transfected cells (Caki/Vec) and Mcl-1-(Caki/Mcl-1) or Bcl-2-overexpressing cells (Caki/Bcl-2) were treated with 20 μM curcumin in the presence or absence of 0.5 μM PP242 for 30 h. Immunoblot analysis of PARP, Mcl-1, Bcl-2, and/or actin. The data represented in **a**, **c**, **d**, **e**, **f**, and **l** are the means ± s.d. from three independent samples. **p *<* 0.01 compared to the control. #*p* *<* 0.01 compared to the combined treatment with PP242 plus curcumin. *p *<* 0.01 compared to the combined treatment with PP242 plus curcumin-treated Caki/Vec

Next, we examined whether natural compounds enhance PP242-induced apoptosis. First, we selected sub-toxic dosage of natural compounds to sensitize PP242-induced apoptosis (Supplementary Fig. 1a). Curcumin markedly induced sub-G1 population in PP242-treated cells at 30 h (Fig. 2a and Supplementary Fig. 1b), and combined curcumin and PP242 treatment induced chromatin damage and DNA fragmentation (Fig. 2b, c). Furthermore, curcumin plus PP242 increased caspase-3 activity (Fig. 2d), and curcumin plus PP242-induced apoptosis was controlled by caspase-3 activation (Fig. 2e).

Since, the loss-of-mitochondrial membrane potential (MMP) plays a pivotal role in apoptosis [25] and Bax plays critical role in the translocation of cytochrome *c* from the mitochondria [26], we elucidated the effect of curcumin and PP242 on MMP and Bax activation. Combined treatment induced the loss-of-MMP (Fig. 2f) and increased cytochrome *c* levels in the cytosolic fractions (Fig. 2g). As shown in Fig. 2h–j, we examined the activation of Bax using conformation-specific anti-Bax antibody (6A7) [27] and the oligomerization of Bax in curcumin plus PP242-treated cells. Combined treatment induced Bax activation (Fig. 2h, i) and Bax oligomerization (Fig. 2j), indicating that the apoptotic effects of PP242 and curcumin co-treatment are dependent on canonical caspase-induced cell death.

To elucidate the molecular mechanisms leading to apoptosis in curcumin plus PP242-treated cells, we analyzed regulation of apoptosis-related proteins expression. As shown Fig. 2k, curcumin plus PP242 altered Mcl-1 and Bcl-2 protein expression. We subsequently investigated the functional importance of two anti-apoptotic proteins. Ectopic overexpression of Mcl-1 or Bcl-2 attenuated curcumin plus PP242-induced apoptosis and cleavage of PARP (Fig. 2l). Therefore, our finding showed that the downregulation of Mcl-1 and Bcl-2 expression is associated with curcumin plus PP242-induced apoptosis.

### Combined curcumin and PP242 treatment does not induce apoptosis in normal cells

**Fig. 3:**
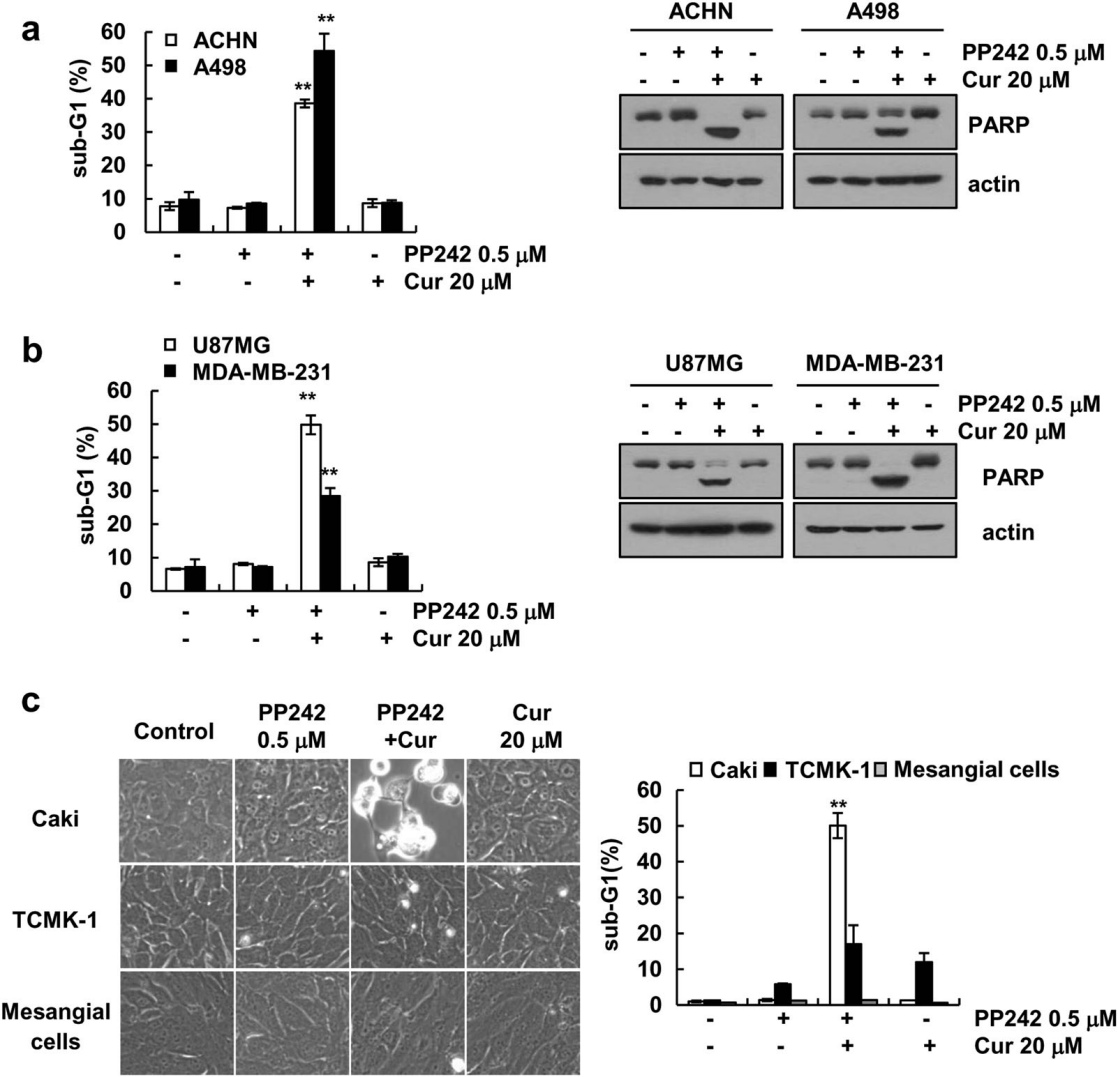
Effects of combined curcumin and PP242 treatment on apoptosis in other carcinoma cell lines and normal cells. **a**–**c** Cells were treated 20 μM curcumin plus 0.5 μM PP242 for 30 h. Immunoblot analysis of PARP and actin. The cell morphology was analyzed using interference light microscopy. The data represented in **a**, **b**, and **c** are the means ± s.d. from three independent samples. **p *<* 0.01 compared to control

Next, we tested whether the anti-cancer effects of combined curcumin and PP242 treatment could be applied to other cells. Combined curcumin and PP242 treatment increased apoptotic population and PARP cleavage in cancer cells (renal carcinoma: ACHN and A498, glioma: U87MG, and breast carcinoma: MDA-MB-231) (Fig. 3a, b), but did not alter cell morphology and sub-G1 population in normal cells (mouse kidney cells: TCMK-1, and normal human mesangial cells) (Fig. 3c). Therefore, our finding supported that curcumin could enhance PP242-mediated apoptosis in multiple cancer cells.

### Attenuation of Rictor and Akt expression plays a critical role in combined curcumin and PP242 treatment-induced apoptosis

**Fig. 4:**
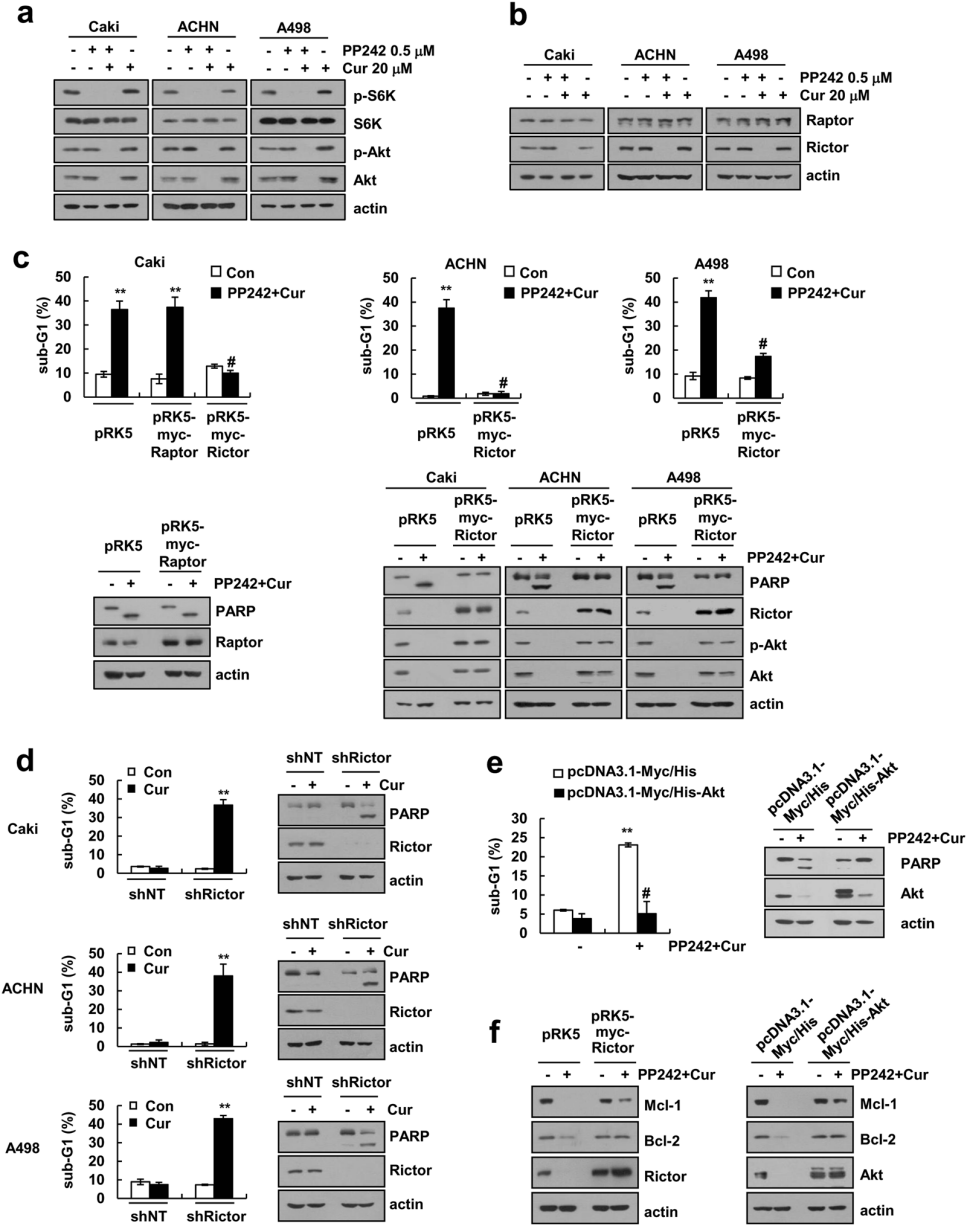
Rictor downregulation contributes to the curcumin plus PP242-induced apoptosis. **a**, **b** Renal carcinoma cells were treated with 20 μM curcumin in the presence or absence of 0.5 μM PP242 for 30 h. Immunoblot analysis of p-S6K, S6K, p-Akt, Akt, Raptor, Rictor, and actin. **c** Caki, ACHN, and A498 cells were transiently transfected with pRK5, pRK5-myc-Raptor, or pRK5-myc-Rictor. After transfection, cells were treated 20 μM curcumin and 0.5 μM PP242 for 30 h. Immunoblot analysis of PARP, Raptor, Rictor, p-Akt, Akt, and actin. **d** Caki, ACHN, and A498 cells were transduced with lentivirus containing either shRNA targeting Rictor or a non-target (NT) sequence. After transduction and selection, cells were treated with 20 μM curcumin for 30 h. Immunoblot analysis of PARP, Rictor, and actin. **e** Caki cells were transiently transfected with pcDNA3.1-myc/His or pcDNA3.1-myc/His-Akt. After transfection, Caki cells were treated with 20 μM curcumin and 0.5 μM PP242 for 30 h. Immunoblot analysis of PARP, Akt, and actin. **f** Caki cells were transiently transfected with pRK5, pRK5-myc-Rictor, pcDNA3.1-myc/His, or pcDNA3.1-myc/His-Akt. After transfection, Caki cells were treated with 20 μM curcumin and 0.5 μM PP242 for 30 h. Immunoblot analysis of Mcl-1, Bcl-2, Rictor, Akt, and actin. The data represented in **c**, **d**, and **e** are the means ± s.d. from three independent samples. **p *<* 0.01 compared to control. #*p* *<* 0.01 compared to curcumin plus PP242-treated vector

PP242 inhibits mTORC1 and mTORC2 signaling pathways. Therefore, we investigated which signaling pathways play critical roles in combined curcumin and PP242 treatment-mediated apoptosis. mTORC1 and mTORC2 phosphorylates S6K and Akt, respectively [1]. PP242 inhibited the phosphorylation of S6K, but the inhibition of Akt phosphorylation was transient (Fig. 1e). As shown in Fig. 4a, PP242 also did not inhibit Akt phosphorylation at 30 h in human renal carcinoma ACHN and A498 cells (Fig. 4a). However, combined curcumin and PP242 treatment maintained the dephosphorylation of Akt (Fig. 4a). Furthermore, combined curcumin and PP242 treatment attenuated the protein levels of Rictor and Akt, but not Raptor (Fig. 4a, b). Therefore, we investigated whether Rictor and Akt play critical roles in curcumin and PP242-induced apoptosis. Ectopic overexpression of Rictor markedly inhibited combined curcumin and PP242 treatment-induced apoptosis and Akt downregulation in human renal carcinoma Caki, ACHN, and A498 cells (Fig. 4c). To further confirm the importance of Rictor on apoptosis, renal carcinoma cells were infected with lentiviruses carrying shNT or shRictor. Curcumin alone markedly induced apoptosis in shRictor-infected cells (Fig. 4d and Supplementary Fig. 2a). Furthermore, the ectopic overexpression of Akt, a downstream signaling kinase of Rictor, also inhibited curcumin plus PP242-induced apoptosis (Fig. 4e). In contrast, curcumin and PP242 did not alter the activation and expression of other downstream signaling kinase of mTORC2 (PKCα and SGK1) (Supplementary Fig. 3a and b). In addtion, ectopic overexpression of Rictor or Akt inhibited the downregulation of Mcl-1 and Bcl-2 expression by combined curcumin and PP242 treatment (Fig. 4f). Therefore, our data indicated that inhibition of the mTORC2-Akt signaling pathway controls curcumin plus PP242-induced apoptosis.

### Combined treatment increases cytosolic calcium levels and induces lysosomal membrane permeabilization, resulting in the induction of autophagy

**Fig. 5:**
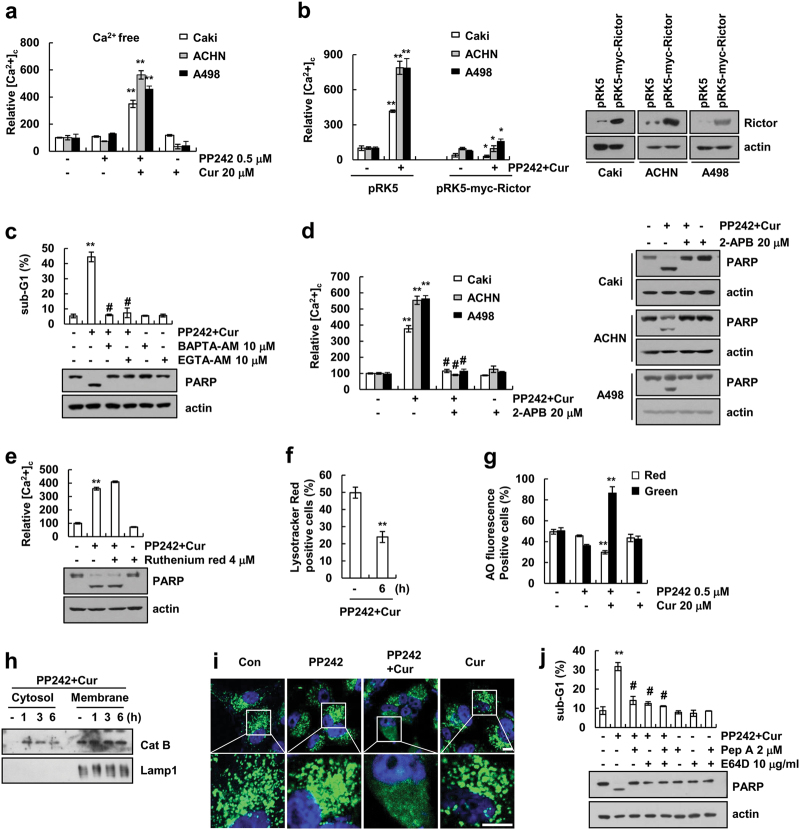
Combined curcumin plus PP242 treatment induces the upregulation of cytosolic calcium levels and lysosomal membrane permeabilization. **a** Caki, ACHN, and A498 cells were treated with 20 μM curcumin in the presence or absence of 0.5 μM PP242 for 6 h. After treatment, the cells were loaded with the Ca^2+^ dye Fluo-4/AM, and calcium levels were detected by flow cytometry. **b** Caki, ACHN, and A498 cells were transiently transfected with pRK5 or pRK5-myc-Rictor. After transfection, cells were treated with 20 μM curcumin and 0.5 μM PP242 for 6 h. The cells were loaded with the Ca^2+^ dye Fluo-4 AM, and calcium levels were detected by flow cytometry. Immunoblot analysis of Rictor and actin. **c** Caki cells were pretreated with 10 μM BAPTA-AM and 10 μM EGTA-AM for 30 min, and then added 20 μM curcumin and 0.5 μM PP242 for 30 h. Immunoblot analysis of PARP and actin. **d**, **e** Renal carcinoma cells were pretreated with 20 μM 2-aminoethosxydiphenyl borate (2-APB) or 4 μM Ruthenium red for 30 min, and then treated with 20 μM curcumin and 0.5 μM PP242 for 6 h or 30 h. The cells were loaded with the Ca^2+^ dye Fluo-4 AM, and calcium levels were detected by flow cytometry. Immunoblot analysis of PARP and actin. **f** Caki cells were treated with 20 μM curcumin and 0.5 μM PP242 for 6 h. After treatment, Caki cells were loaded with LysoTracker Red fluorescent dye. The fluorescence intensity was detected by flow cytometry. **g** Caki cells were incubated with 5 μg/ml acridine orange (AO) for 15 min, and then treated with 20 μM curcumin in the presence or absence of 0.5 μM PP242 for 6 h. After treatment, the fluorescence intensity was detected by flow cytometry. **h** Caki cells were treated with 20 μM curcumin and 0.5 μM PP242 for the indicated time periods. After treatment, cytosol and membrane fractions (lysosome-rich fraction) were prepared. Immunoblot analysis of cathepsin B (Cat B) and Lamp1. **i** Cells were incubated for 2 h with 5 mg/ml 10 kDa FITC-dextran for 3 h. After a 2 h chasing period, cells were treated with 20 μM curcumin in the presence or absence of 0.5 μM PP242 for 6 h. FITC-dextran was observed by confocal microscope. Scale bar = 10 μm. **j** Caki cells were pretreated with 2 μM pepstatin A (Pep A) and/or 10 μg/ml E64D for 30 min, and then added with 20 μM curcumin and 0.5 μM PP242 for 30 h. Immunoblot analysis of PARP and actin. The data represented in **a**–**g**, and **j** are the means ± s.d. from three independent samples. **p *<* 0.01 compared to control. *p *<* 0.01 compared to curcumin plus PP242-treated vector. #*p* *<* 0.01 compared to curcumin plus PP242

Previous studies have reported that Rictor is mainly located in the ER [28], and defects in Rictor and/or Akt induce cytosolic calcium release [29, 30]. Therefore, we investigated whether PP242 plus curcumin increases cytosolic calcium levels. As shown in Fig. 5a, single treatment had no effect on cytosolic calcium levels, but combined PP242 and curcumin treatment markedly increased calcium levels within 6 h. Furthermore, ectopic overexpression of Rictor inhibited the increase of cytosolic calcium levels (Fig. 5b). To investigate the effect of the upregulated calcium concentration on apoptosis, the cells were treated with calcium chelators (BAPTA-AM and EGTA-AM). Both chelators significantly inhibited the apoptosis and cleavage of PARP in PP242 plus curcumin-treated cells (Fig. 5c). These data indicated that cytosolic calcium concentration plays critical roles on PP242 plus curcumin-induced apoptosis. Next, we investigated the possibility the mitochondria and/or ER as a source of calcium release using Ruthenium red (inhibitor of mitochondrial Ca^2+^ uniporter) and 2-APB (blocker of the inositol 1,4,5-trisphosphate (IP3)-induced Ca^2+^ release), respectively. 2-APB markedly inhibited cytosolic calcium increase and apoptosis, but not Ruthenium red (Fig. 5d, e). In addition, increased intracellular calcium induced lysosomal membrane potential (LMP) involved in cell death in cancer cells [31, 32]. As shown in Fig. 5f–i, we investigate whether curcumin plus PP242-induced LMP. Combined treatment with curcumin plus PP242 reduced LysoTracker Red-positive lysosomes in cells (Fig. 5f). Acridine Orange (AO) accumulates in lysosomes, resulting the induction of red fluorescence, but LMP induces the translocation of AO from the lysosomes to cytosol, resulting in the induction of green fluorescence [33]. Combined curcumin and PP242 treatment decreased red fluorescence, but increased green fluorescence (Fig. 5g). Furthermore, curcumin plus PP242-induced the release of cathepsin B and FITC-dextran into the cytosol (Fig. 5h, i). In addition, inhibitors of cathepsins (pepstatin A and E64D) markedly inhibited PP242 plus curcumin-induced apoptosis (Fig. 5j), suggesting that the apoptotic effects of PP242 and curcumin depend on the cytosolic release of lysosomal cathepsins.

**Fig. 6:**
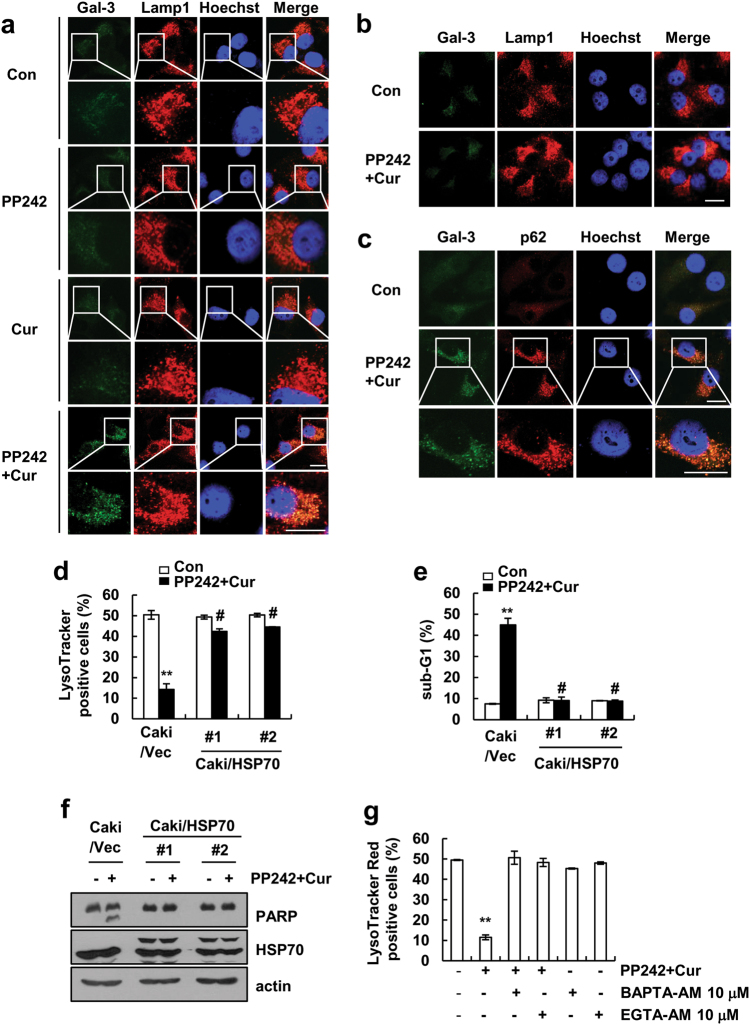
Combined treatment with curcumin plus PP242 induces autophagy. **a**, **b** Caki cells were treated with 20 μM curcumin and 0.5 μM PP242 for 6 h (**a**) or 24 h (**b**). After treatment, cells were fixed and subjected to immunocytochemistry for Galectin-3 (green), Lamp1 (red), and DAPI (blue). Scale bar = 20 μm. **c** Caki cells were treated with 20 μM curcumin and 0.5 μM PP242 for 6 h. After treatment, cells were fixed and subjected to immunocytochemistry for Galectin-3 (green), p62 (red), and DAPI (blue). Scale bar = 20 μm. **d**–**f** Vector-transfected cells (Caki/Vec) and HSP70 (Caki/HSP70)-overexpressing cells were treated with 20 μM curcumin and 0.5 μM PP242 for 6 h (**d**) or 30 h (**e** and **f**) and then loaded with LysoTracker Red fluorescent dye. The fluorescence intensity was detected by flow cytometry (**d**). The level of apoptosis was assessed by measuring the sub-G1 fraction using flow cytometry (**e**). Immunoblot analysis of PARP, HSP70 and actin (**f**). **g** Caki cells were with 10 μM BAPTA-AM and 10 μM EGTA-AM for 30 min, and then added with 20 μM curcumin and 0.5 μM PP242 for 6 h. After treatment, cells loaded with LysoTracker Red fluorescent dye. The fluorescence intensity was detected by flow cytometry. The data represented in **d**, **e**, and **g** are the means ± s.d. from three independent samples. **p *<* 0.01 compared to control. #*p* *<* 0.01 compared to curcumin plus PP242-treated Caki/Vec

When lysosomes undergo LMP resulting in a local increase of pH in the cytosol, the induction of autophagy of lysosome (lysophagy) is also observed [34]. Lysosomal damage triggers the autophagic response, resulting in the removal of damaged lysosomes [35]. Therefore, we investigated whether combined PP242 and curcumin treatment induces autophagy. First, since galectin-3, normally localized to the cytoplasm and nucleus, accumulates in damaged lysosomes [35], we examined the recruitment of galectin-3 to lysosomes in PP242 plus curcumin-treated cells. As shown in Fig. 6a, galectin-3 puncta co-localized with Lamp1 in curcumin and PP242-treated cells at 6 h, but not in cells treated with PP242 alone or curcumin alone. However, we did not detect the galectin-3 puncta at 24 h (Fig. 6b). Lysophagy also uses autophagy machinery, such as p62 and LC3 [36]. Galectin-3-positive damaged lysosomes were co-localized with p62 in curcumin plus PP242-treated cells (Fig. 6c).

To further investigate the importance of combined treatment-induced LMP, we used HSP70-overexpressing cells, which inhibit the induction of LMP [37]. Ectopic expression of HSP70 markedly inhibited LMP (Fig. 6d), and apoptosis in PP242 plus curcumin-treated cells (Fig. 6e, f). Next, we investigated whether increased cytosolic calcium levels cause LMP induction. Both BAPTA-AM and EGTA-AM, calcium chelators, remarkably inhibited PP242 plus curcumin-induced LMP (Fig. 6g). Therefore, our findings indicated that combined curcumin and PP242 treatment induces apoptosis through the induction of cytosolic calcium-mediated LMP.

### Combined PP242 and curcumin treatment induces autophagy-mediated apoptosis

**Fig. 7:**
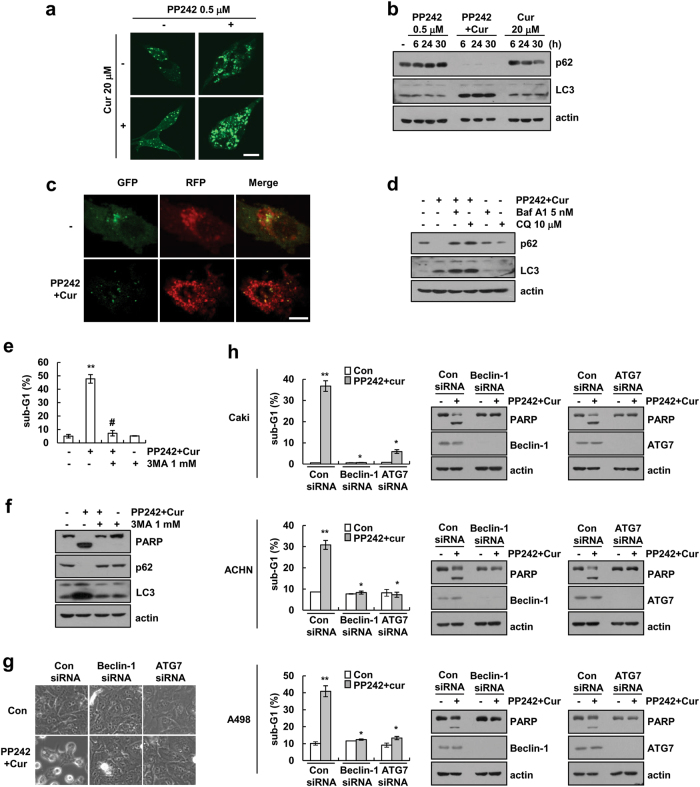
Combined curcumin plus PP242 treatment induces autophagy flux. **a** Caki/LC3 cells were treated with 20 μM curcumin in the presence or absence of 0.5 μM PP242 for 24 h. GFP-LC3 puncta were observed by confocal microscope. Scale bar = 10 μm. **b** Caki cells were treated with 20 μM curcumin in the presence or absence of 0.5 μM PP242 for the indicated time periods. Immunoblot analysis of p62, LC3, and actin. **c** Caki cells were transiently transfected with mRFP-EGFP-LC3 and then treated with 20 μM curcumin plus 0.5 μM PP242 for 24 h. mRFP-EGFP-LC3 puncta were observed by confocal microscope. Scale bar = 10 μm. **d** Caki cells were pretreated with 10 μM chloroquine (CQ) and 5 nM bafilomycin A1 (Baf A1) for 30 min, and then added with 20 μM curcumin plus 0.5 μM PP242 for 24 h. Immunoblot analysis of LC3, p62 and actin. **e**, **f** Caki cells were pretreated with 1 mM 3-methyladenine (3-MA) for 30 min, and then added with 20 μM curcumin plus 0.5 μM PP242 for 30 h. Immunoblot analysis of PARP, p62, LC3, and actin (**f**). **g**–**h** Renal carcinoma cells were transiently transfected with control siRNA (Con siRNA), beclin-1 siRNA, or ATG7 siRNA. After transfection, Caki cells were treated with 20 μM curcumin plus 0.5 μM PP242 for 30 h. The cell morphology was examined using interference light microscopy (**g**). Immunoblot analysis of PARP, beclin-1, ATG7 and actin (**h**). The data represented in **e** and **h** are the means ± s.d. from three independent samples. **p *<* 0.01 compared to control. #*p* *<* 0.01 compared to curcumin plus PP242. *p *<* 0.01 compared to curcumin plus PP242-treated control siRNA

Since damaged lysosomes are removed by autophagy and autophagy promotes or inhibits cell death, depending on cancer initiation or maintenance, respectively [38–40], we investigated the role of autophagy on PP242 plus curcumin-induced apoptosis. PP242 plus curcumin markedly induced LC3 puncta, the downregulation of p62 expression, and the upregulation of LC3 II levels, but these effects were not observed with PP242 and curcumin treatment alone (Fig. 7a, b). To detect induction of autophagy flux, we used mRFP-EGFP-LC3 plasmids. Combined PP242 and curcumin treatment increased the number of RFP positive and GFP negative puncta at 24 h (Fig. 7c), and chloroquine (CQ) and bafilomycin A1 (Baf A1) markedly increased LC3 II levels by curcumin plus PP242 (Fig. 7d). Therefore, PP242 and curcumin increased autolysosome formation via the fusion of autophagosomes and lysosomes. Next, to test the effect of autophagy on apoptosis, Caki cells were treated with 3-Methyladenine (3-MA), an autophagy inhibitor. 3-MA markedly inhibited apoptosis by combined PP242 and curcumin treatment (Fig. 7e, f). 3-MA could activate autophagy though the inhibition of PI3K [41], thus, we further confirmed the effect of autophagy using siRNA of ATG7 and beclin-1. The downregulation of ATG7 and beclin-1 also prevented the combined treatment-induced apoptosis in human renal carcinoma Caki, ACHN, and A498 cells (Fig. 7g, h, and Supplementary Fig. 2b). Therefore, our data suggested that PP242 plus curcumin enhance apoptosis via induction of autophagy.

### Combined PP242 and curcumin treatment inhibits tumor growth and induces cell death in vivo

**Fig. 8:**
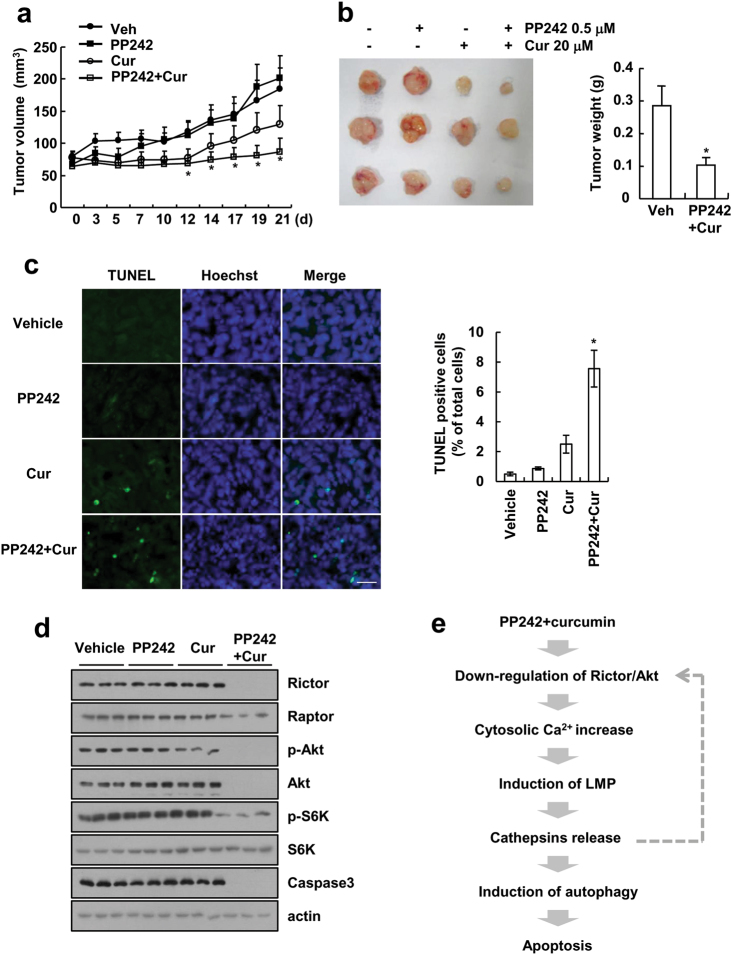
Tumor growth in vivo is reduced by combined curcumin plus PP242 treatment. Tumor volume was monitored during treatments: vehicle, PP242 (20 mg/kg; i.p.), curcumin (50 mg/kg; i.p), or PP242 plus curcumin for 21 days. **a** The graph shows changes in the tumor volume. Number of animals per group = 7. The data represented are the means ± SEM (*n* = 7). **b** The size and weight of the dissected tumors are shown. **c** Representative images of tumor sections that were analyzed by TUNEL assay. Nuclear staining was performed with Hoechst. Scale bar = 20 μm. TUNEL-positive cells were quantified by counting nuclei in five randomly chosen fields. The values in **c** represent the means ± s.d. **d** Immunoblot analysis of Rictor, Raptor, p-Akt, Akt, p-S6K, S6K, caspase-3, and actin. **e** The scheme showing the mechanism of curcumin plus PP242-induced apoptosis. *p *<* 0.05 compared to vehicle

Next, we analyzed the anti-cancer effect of combined PP242 and curcumin treatment using an in vivo xenograft model. Tumor-bearing mice were treated with PP242 alone, curcumin alone, and PP242 combined with curcumin. Combined PP242 and curcumin treatment markedly inhibited tumor growth, compared with that of vehicle and single treatment (Fig. 8a, b). Furthermore, tumor weight was markedly reduced by treatment with PP242 plus curcumin (Fig. 8b). In addition, we also detected cell death using TUNEL analysis, and PP242 plus curcumin increased TUNEL-positive cells (Fig. 8c). Finally, we investigated the effect of PP242 and/or curcumin on the mTORC1/2 signaling pathway in vivo. Combined PP242 and curcumin treatment attenuated Rictor expression and induced dephosphorylation of Akt and S6K (Fig. 8d). However, the expression of Raptor was not changed by treatment with PP242 plus curcumin (Fig. 8d). These data indicated that combined PP242 and curcumin treatment inhibits tumor growth and induces apoptosis in vivo.

## Discussion

Our data demonstrated that combined treatment decreased expression of Rictor and Akt protein levels, which then increased cytosolic Ca^2+^ levels. Increased Ca^2+^ in turn induced LMP, and damaged lysosomes activated a signaling cascade of autophagy, resulting in autophagy of lysosome (lysophagy). PP242 plus curcumin-mediated autophagy induced apoptosis (Fig. 8e). In addition, we found that combined PP242 and curcumin treatment induced apoptosis in cancer cells, but not in normal cells. Furthermore, combined with PP242 and curcumin treatment also had anti-cancer effects, such as reduction of tumor size and induction of cell death in xenograft models.

The rapamycin analog only inhibits mTORC1, and the chronic inhibition of mTORC1 impedes the negative feedback loop, followed by activation of PI3K and Akt. Therefore, to complement the limitation of the rapamycin analog, inhibitors of mTORC1/2 or dual PI3K-mTOR1/2 have been developed. However, PI3K has multiple functions, including normal cell survival, metabolism and differentiation, and thus, the inhibition of PI3K-mTORC1/2 could induce mTORC1/2-independent events [42, 43]. Among inhibitors of mTORC1/2 inhibitors, PP242-induced inhibition of S6K phosphorylation was maintained up to 30 h, but Akt dephosphorylation was reversed (Fig. 1e). In a previous study, PP242 transiently attenuates the mTORC2/Akt activity in colorectal carcinoma cells [44]. Wang et al.[44] reported that PP242 increased the phosphorylation of EGFR, resulting in the upregulation of Akt phosphorylation. In addition, PP242 also increased the phosphorylation of ERK in multiple myeloma cells [5]. PP242 stimulated RAF activation, and then increased ERK phosphorylation, thus combined treatment with ERK inhibitor (U0126) and PP242 increased cell death [5]. The mTORC1 is predominantly phosphorylated on mTOR (Ser2448), whereas mTORC2 is predominantly phosphorylated on mTOR (Ser2481) [45]. Interestingly, the phosphorylation of both mTOR proteins was reduced by PP242 treatment until 30 h (Fig. 1e). In our study, curcumin plus PP242 inhibited phosphorylation of Akt until 30 h and induced the downregulation of Rictor and Akt protein levels (Fig. 4a, b). Ectopic expression of Rictor and Akt inhibited apoptosis and dephosphorylation of Akt (Fig. 4c, e). In contrast, curcumin plus PP242 also induced the downregulation of Sin1 and PRR5, which are other components of mTORC2 (Supplementary Fig. 4a, b). However, ectopic overexpression of Sin1, PRR5 and PRR5L had no effect on apoptosis and attenuation of Mcl-1 and Bcl-2 expression by combined curcumin and PP242 treatment (Supplementary Fig. 4c). In addition, Beevers et al. [46] reported that curcumin disrupts the association of mTOR with Raptor and at higher levels the binding of mTOR with Rictor in IGF-1-treated conditions. However, each treatment with PP242 and curcumin did not change the association of mTOR with Raptor and Rictor in our conditions (Supplementary Fig. 5). Furthermore, combined treatment with PP242 and curcumin also has no effect on binding of the mTOR and Raptor. However, combined treatment with PP242 and curcumin inhibited association of the mTOR and Rictor at 30 h (Supplementary Fig. 5). We think that the downregulation of Rictor might affect the formation of the complex between mTOR and Rictor in PP242 plus curcumin-treated cells. Therefore, the downregulation of Rictor-Akt expression is important for curcumin plus PP242-induced apoptosis.

We also found that curcumin plus PP242 did not inhibit Rictor and Akt mRNA expression (Supplementary Fig. 6a and 6b), thus we assessed the effect of the proteasome and lysosome on the downregulation of Rictor expression. Both inhibitors of proteasome and lysosome did not affect the downregulation of Rictor (Supplementary Fig. 6c and 6d), but cathepsin inhibitors rescued the downregulation of Rictor protein levels in curcumin and PP242-treated cells (Supplementary Fig. 6e). We suggested that amplification loop (Rictor downregulation, calcium release, and LMP-mediating cathepsin release) might be involved in curcumin plus PP242-mediated apoptosis (Fig. 8e). However, further experiments are needed to identify how cathepsin reduces Rictor protein levels.

When autophagy occurs by a variety of conditions, phagophore sequesters cytoplasmic components, including organelles, to form autophagosomes, which fuse with the lysosome to form autolysosome-degrading luminal contents. This degradation is important for the maintenance of cellular homeostasis. The selective autophagy of organelles is also critical for cellular homeostasis. Recently, several studies have reported organelle-specific autophagy, including mitophagy [47], pexophagy [48], reticulophagy [49], and nucleophagy [50]. The lysosome also undergoes autophagy to control the number and quality of this organelle, a process referred to as lysophagy. Hung et al. [34] reported that the ubiquitination of damaged lysosomes by light recruits autophagy-related proteins and is crucial for the formation of autolysosome. In addition, the lysosomes damaged by lysosomotropic reagents (l-Leucyl-l-leucine methyl ester) are selectively removed by autophagy, and this lysophagy plays critical roles in the suppression of acute kidney injury in vivo, due to reduced lysosomal rupture-induced damage [35]. Although damaged lysosomes lack digestive ability, the total number of lysosomes does not alter [35]. Therefore, if damaged lysosomes are not removed, activity of cellular lysosomal activity is reduced, and this effect could promote cellular damage. In the present study, galectin-3 puncta were observed at 6 h, but not at 24 h in curcumin plus PP242-treated cells (Fig. 6a, b). Combined treatment-induced damaged lysosomes might be removed by autophagy within 24 h. Nevertheless, the activation of autophagy is associated with induction of cell death (Fig. 7e–h).

Largely, autophagy is considered a survival process, but autophagy also induces cell death, namely, autophagic cell death. For example, 3-decylcatechol induces p62 transcription through the IRE1α/JNK/c-Jun pathway and increased autophagy flux, thereby promoting cell death [39]. Cannabinoid also promotes autophagic cell death through the induction of ER stress [38]. However, many researchers use terms, such as “autophagic cell death”, thus the role of autophagy in cell death is confusing. Shen et al.[51] suggested the definition of autophagic cell death. First, cell death occurs without the involvement of the apoptosis machinery. Second, autophagic flux is induced in cell death. Finally, pharmacological inhibitors and ATG siRNA knockdown is able to rescue or prevent cell death [51]. In this study, PP242 and curcumin increased autophagy flux (Fig. 7d), and autophagy inhibitor (3-MA) and knockdown of beclin-1 and ATG7 rescued combined treatment-induced cell death (Fig. 7e–h). However, we detected caspase activity and caspase inhibitor markedly reduced cell death in curcumin and PP242-treated cells (Fig. 2d, e). Therefore, although curcumin and PP242 increased autophagic flux, this cell death mode is autophagy-mediated apoptosis, not autophagic cell death.

Taken together, our finding suggests that curcumin and PP242 induced autophagy-mediated apoptosis through the cytosolic Ca^2+^-mediated induction of LMP by the downregulation of Rictor expression. Therefore, curcumin might overcome PP242 resistance in cancer cells. Combinatorial treatment with PP242 and curcumin might be a novel and effective therapeutic strategy to fight against various cancers.

## Materials and methods

### Cell culture and materials

ATCC supplied all human cancer cells and normal mouse kidney (TCMK-1) cells (Manassas, VA), and Lonza supplied normal human mesangial cells (Basel, Switzerland). All cell lines tested negative for mycoplasma contamination. The lines were authenticated by standard morphologic examination using microscopy. Cells were cultured in DMEM containing 10% FBS and 100 μg/mL gentamycin. R&D system supplied z-VAD-fmk and TNF-α (Minneapolis, MN), and Biomol supplied curcumin (Plymouth Meeting, PA). LKT Labs (St. Paul, MN) and Selleckchem (Huston, TX) supplied Kahweol and PP242, respectively. Calbiochem supplied Quercetin, Luteolin, 2-aminoethosxydiphenyl borate (2-APB), and EGTA-AM (San Diego, CA). Enzo Life Sciences (Plymouth Meeting, PA) and Cayman Chemical (Ann Arbor, MI) supplied pepstatin A and E64D and, respectively. Sigma Chemical Co. supplied other chemicals (St. Louis, MO). We provided information for used antibodies in Supplementary Table 1. Human Akt1 cDNA (Upstate Biotechnology, Lake Placid, NY) was cloned into pcDNA3.1-Myc/His vector. pRK5 vector was purchased from Clontech Laboratories, Inc. (Mountain View, CA). pRK5-myc-Raptor (Addgene plasmid # 1859) and pRK5-myc-Rictor (Addgene plasmid # 1859) were a gift from David Sabatini [52]. Santa Cruz Biotechnology supplied the siRNA (Santa Cruz, CA).

### FACS analysis for detection of cell death

Detection of sub-G1 population was the same as described previously [53].

### Western blot analysis

Using whole-cell lysis buffer (modified RIPA), lysates were collected and added with 5× SDS loading buffer [53–55]. After separation by SDS-PAGE, proteins were blotted with specific antibodies. The antibodies were detected by ECL (enhanced chemiluminescence) solution, and EMD Millipore supplied ECL kit (Darmstadt, Germany).

### Detection of cell viability, Condensed or fragmented chromatin, and caspase-3 activity

Viability of cells, condensed chromatin and fragmented chromatin, and caspase-3 activity were measured as previously described [53, 56].

### Detection of MMP, cytochrome *c* release, and Bax activation

Detection of mitochondrial membrane potential (MMP), cytochrome *c* release, and Bax activation were the same as described previously [57].

### Stable transfection in Caki cell

pMAX-Bcl-2, pFLAG-CMV4-Mcl-1, pEGFP-HSP70, pEGFP-C3, or pFLAG-CMV4 vector plasmids used for our study. Plasmids were transfected using LipofectAMINE2000 for 2 days, cells were selected by the G418 (700 μg/ml) (Invitrogen, Carlsbad, CA). After 3 weeks, expression of Bcl-2, Mcl-1, and HSP70 was analyzed by western blot in the pooled Caki/vector (vec), Caki/Bcl-2, Caki/Mcl-1, and Caki/HSP70 clones. pEGFP-HSP70 was a gift from Lois Greene (Addgene plasmid # 15215) [58], and pMAX-Bcl-2 was kindly provided by the Dr Rakesh Srivastava. Clontech supplied pEGFP-C3 (Mountain View, CA).

### Intracellular Ca^2+^ detection

Cells were incubated with PBS containing Ca^2+^-sensitive dye Fluo‐4/AM (2 μM) at 37 °C for 45 min, and then cells were resuspended in PBS. Fluorescence was analyzed by flow cytometer. Invitrogen supplied Fluo-4/AM (Carlsbad, CA).

### Measurement of LMP

To monitor of lysosomal destabilization, we used LysoTracker Red, acridine orange (AO), and FITC-dextran. Cells were stained with LysoTracker Red (2.5 μM) or AO (5 μg/ml). Cells were washed twice with PBS, and fluorescence was analyzed by flow cytometer. Using 10 kDa FITC-dextran, 5 mg/ml FITC-dextran was added into the cells at 37 °C for 2 h. After washing with PBS, cells were chased with culture medium for 2 h, and then treated with drugs. Hoechst 33342 (5 μg/ml) added into the cells, and fluorescence was captured by a confocal microscope (Zeiss, New York, NY). Invitrogen (Carlsbad, CA) and Molecular Probes Inc. (Eugene, OR) supplied LysoTracker and AO, respectively. Sigma Chemical Co. supplied 10 kDa FITC-dextran (St. Louis, MO).

### Detection of cathepsin release

Detection of cathepsin release was measured as previously described [59].

### Immunocytochemistry

Cells were fixed by 4% formaldehyde at 4 °C for 15 min, and were induced permeabilization by 50 μg/ml digitonin for 10 min. After blocking with 0.1% gelatin in PBS, cells were stained with corresponding primary antibodies at 4 °C for overnight. After removing the unattached antibodies, and incubated with secondary antibodies [Alexa Fluor (AF) 488- or AF555] at the room temperature for 60 min. To detect nuclei, Hoechst 33342 (5 μg/ml) added into the cells, and then cells were mounted with Prolong Gold. Fluorescence was captured by confocal microscope. Dilutions for primary antibodies were as follows: galectin-3 (sc-23938, 1:100), Lamp1 (sc-5570, 1:200), and p62 (sc-28359, 1:200). Santa Cruz Biotechnology supplied primary antibodies (Santa Cruz, CA), and Life Technologies supplied secondary antibodies and Prolong Gold (Gaithersburg, MD).

### Lentiviral transduction

Non-targeting shRNA or Rictor-targeting shRNA with the pMD2.G and pPsAX2.0 using were introduced to HEK293TN cells TransIT-X2^TM^ Dynamic Delivary System (Mirus Bio LLC, Madison, WI). After 2 days, Caki cells were infected with the filtered lentiviruses containing medium with 5 μg/ml polybrene. We selected transduced cells using 10 μg/ml puromycin for 2 days.

### GFP-LC3 and mRFP-EGFP-LC3 puncta

Cells were transfected with pEGFP-C1-LC3 plasmid using Lipofectamine^TM^ 2000 and cells were selected by 700 μg/ml G418. Caki cells were transiently transfected with mRFP-EGFP-LC3 using Lipofectamine^TM^ 2000. After drugs treatment, cells were mounted with Prolong Gold. Fluorescence signals were captured by confocal microscope.

### In vivo xenograft model

Five-week-old athymic male BALB/c nude mice were provided by Central Lab Animal Inc. (Seoul, Korea). Our protocols were approved by the IRB Keimyung University Ethics Committee (#KM-2014-12). Before starting the experiments, mice were maintained for several days in the temperature and humidity controlled condition. Caki cells (2 × 10^6^) were subcutaneously (s.c.) grafted mice for 2 weeks. A total of 28 mice were randomly divided into four groups and drugs were injected three times a week intraperitoneally: vehicle alone, 20 mg/kg PP242 (20% DMSO + 40% polyethylene-glycol400 in PBS) alone, 50 mg/kg curcumin (20% DMSO + 40% polyethylene-glycol400 in PBS) alone, and PP242 plus curcumin. The size of tumor was determined using Vernier calipers (Mytutoyo Co., Tokyo, Japan), and calculated according to the (length × width^2^)/2. Tumor lysates obtained using RIPA lysis buffer for western blot and tumors were fixed by 30% formalin for overnight for immunostain. Any animals were not excluded from the analysis.

### TUNEL assay

TUNEL staining was the same as described previously [60].

### Statistical analysis

We repeated experiments in our studies at least three times, and all data are represented as the means. Statistical analysis was performed by a one-way ANOVA and post hoc comparisons (Student-Newman–Keuls) using the SPSS (Statistical Package for the Social Sciences, version 22.0) (SPSS Inc.; Chicago, IL). We decide the sample size on the basis of the minimum effects we wish to measure. The *p*-values <0.05 were considered significant.

## Electronic supplementary material

Supplementary Fig.1

Supplementary Fig.2

Supplementary Fig.3

Supplementary Fig.4

Supplementary Fig.5

Supplementary Fig.6

Supplementary infomation

Supplementary table 1
