# Supplementary figures and images for: mTORC1/2 inhibitor and curcumin induce apoptosis through lysosomal membrane permeabilization-mediated autophagy
Source: Oncogene. 2018 May 30;37(38):5205–20. doi: 10.1038/s41388-018-0345-6 (PMC6147804; doi:10.1038/s41388-018-0345-6)

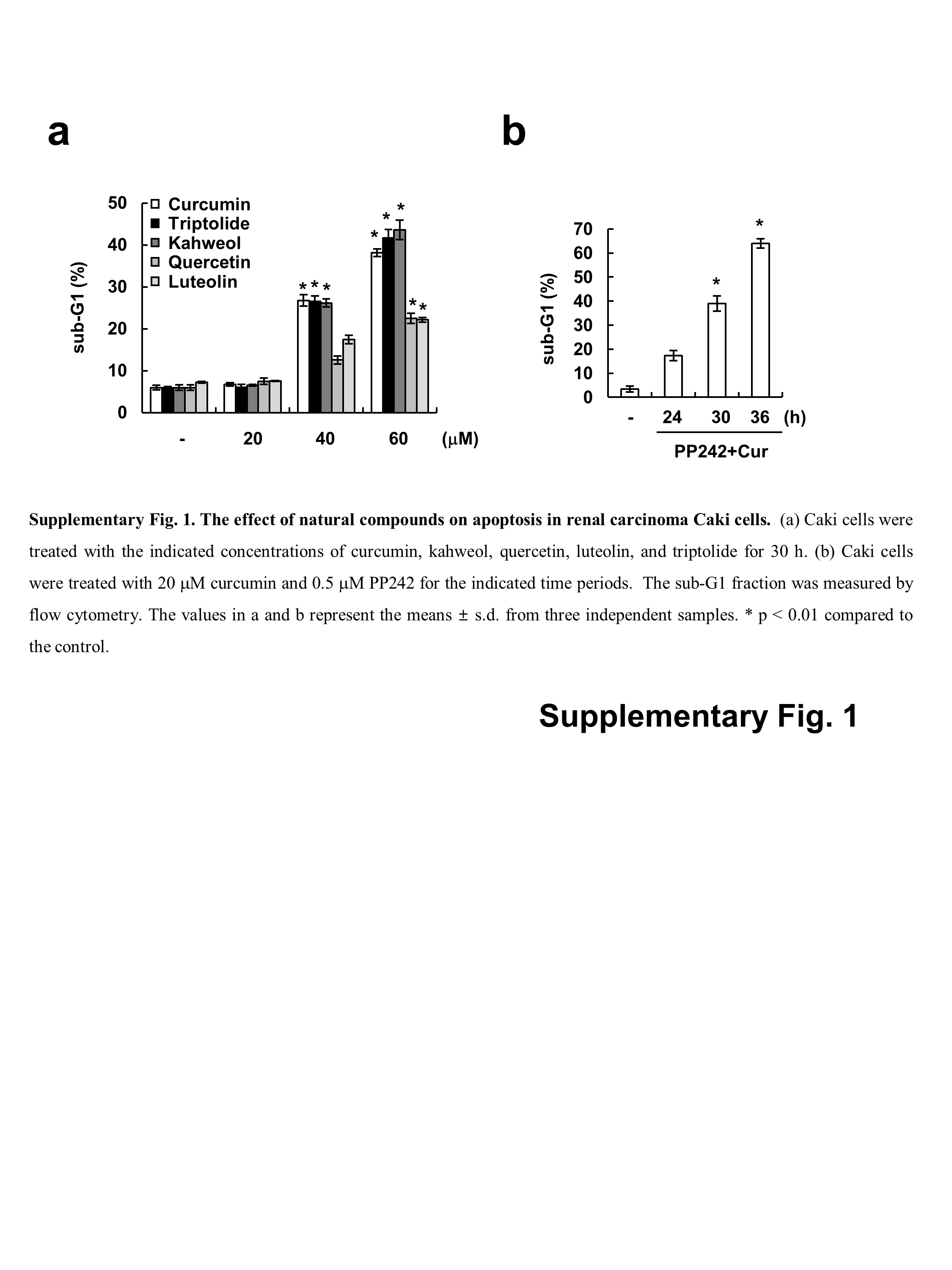

Supplement: Supplementary file 1 — Supplementary Fig.1 [file 41388_2018_345_MOESM1_ESM.tif]

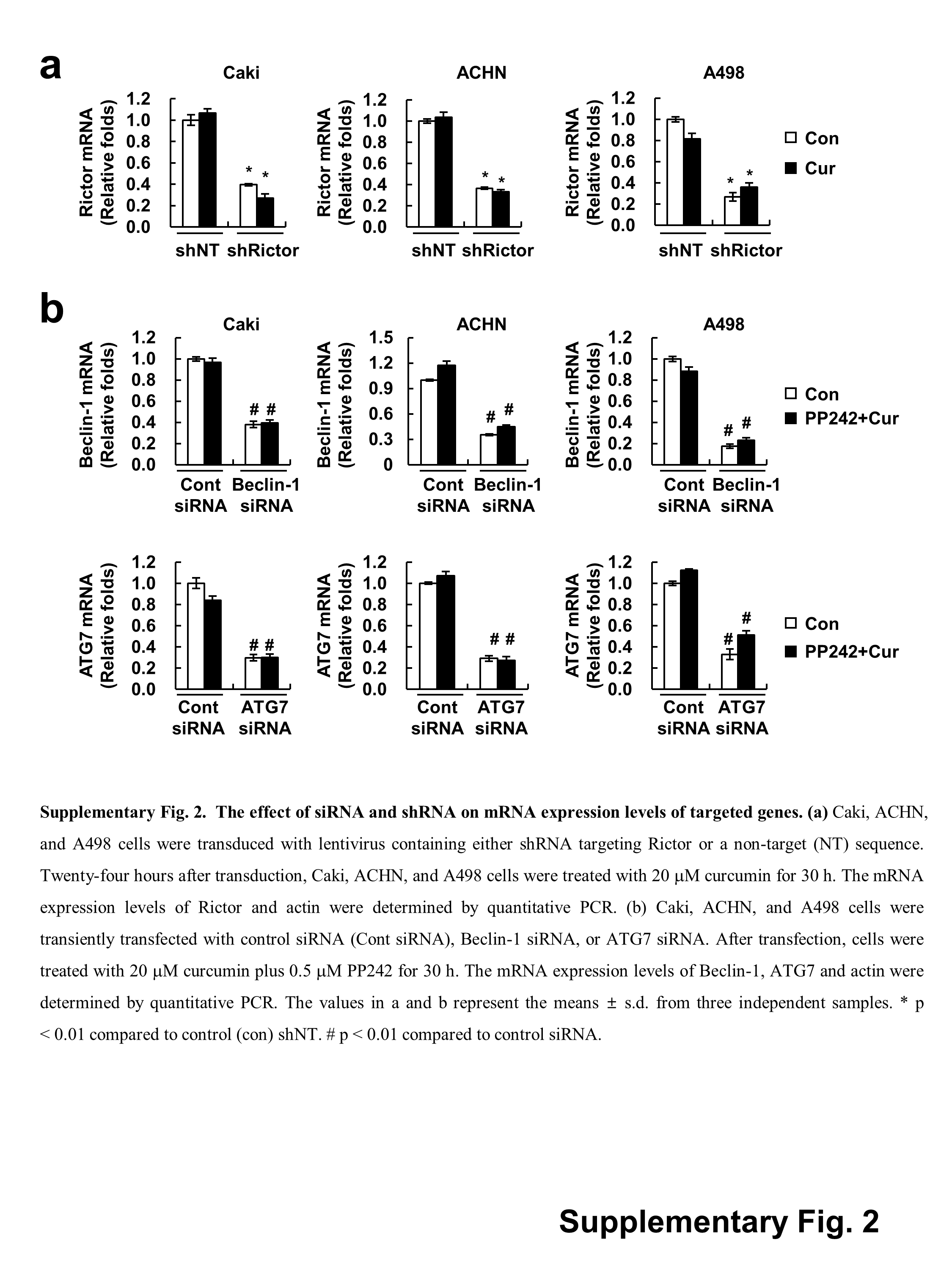

Supplement: Supplementary file 2 — Supplementary Fig.2 [file 41388_2018_345_MOESM2_ESM.tif]

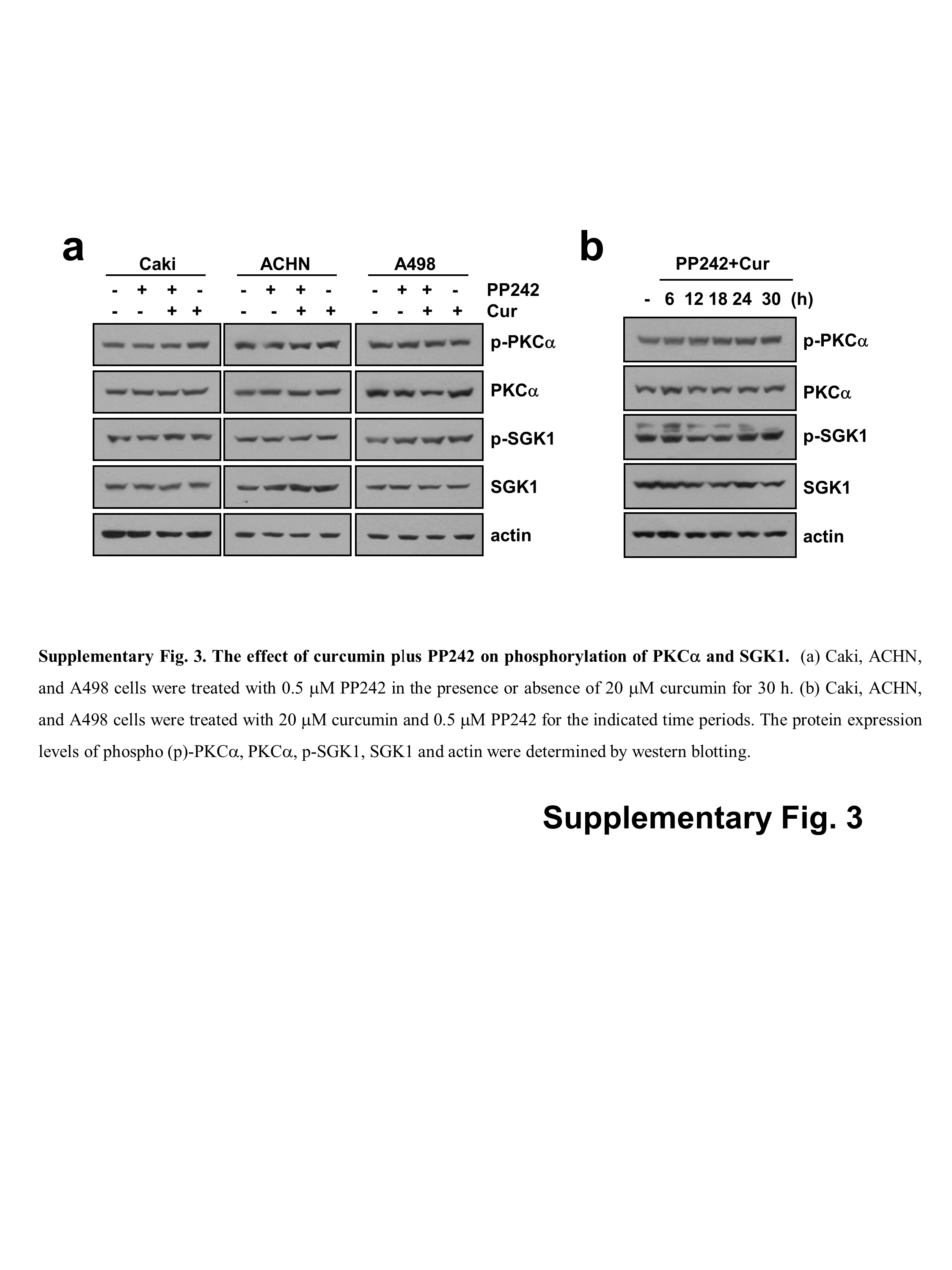

Supplement: Supplementary file 3 — Supplementary Fig.3 [file 41388_2018_345_MOESM3_ESM.tif]

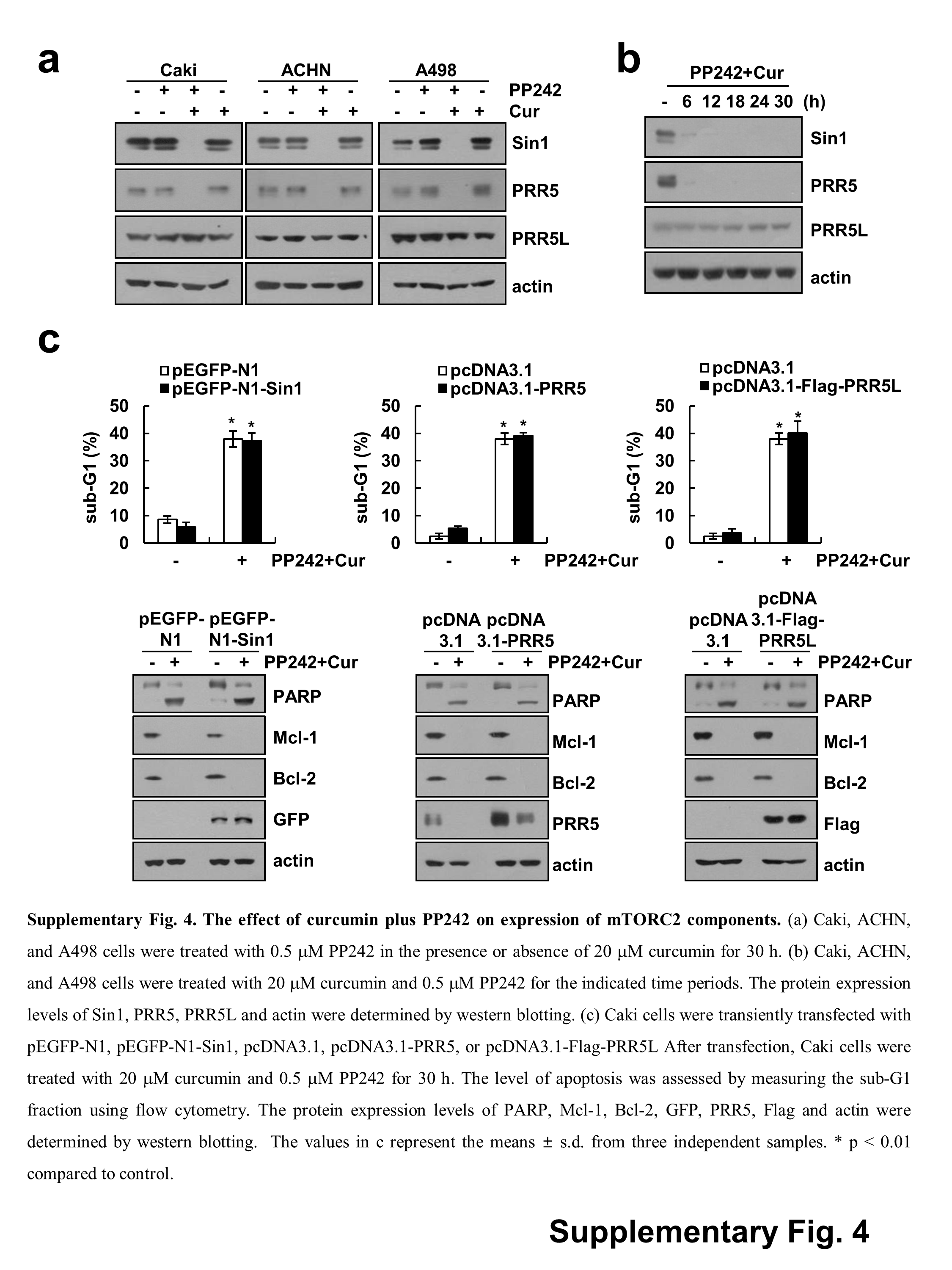

Supplement: Supplementary file 4 — Supplementary Fig.4 [file 41388_2018_345_MOESM4_ESM.tif]

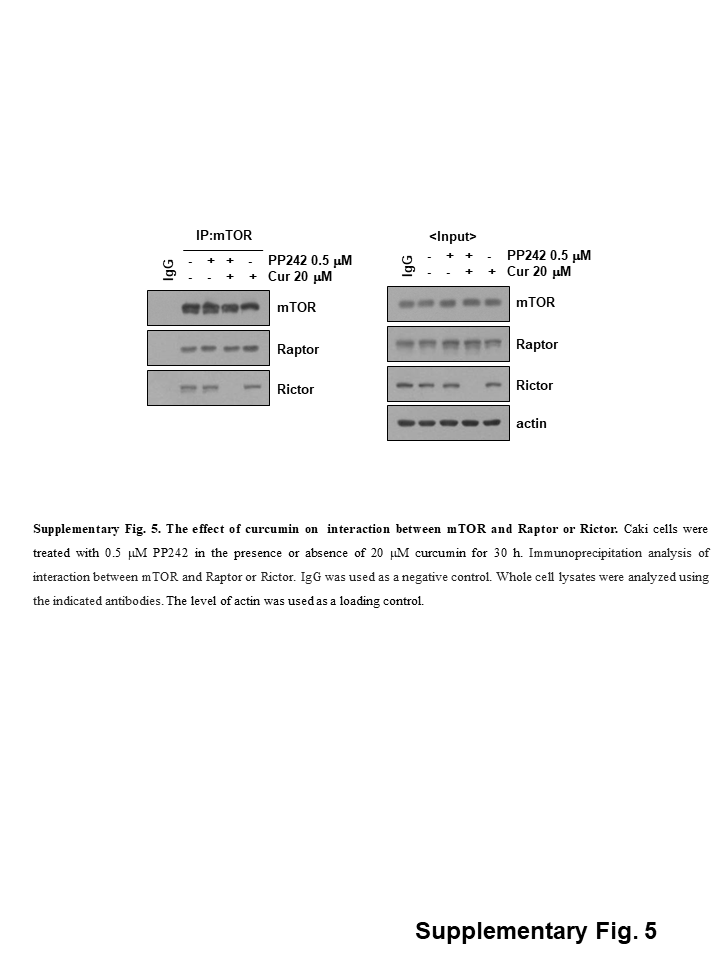

Supplement: Supplementary file 5 — Supplementary Fig.5 [file 41388_2018_345_MOESM5_ESM.tif]

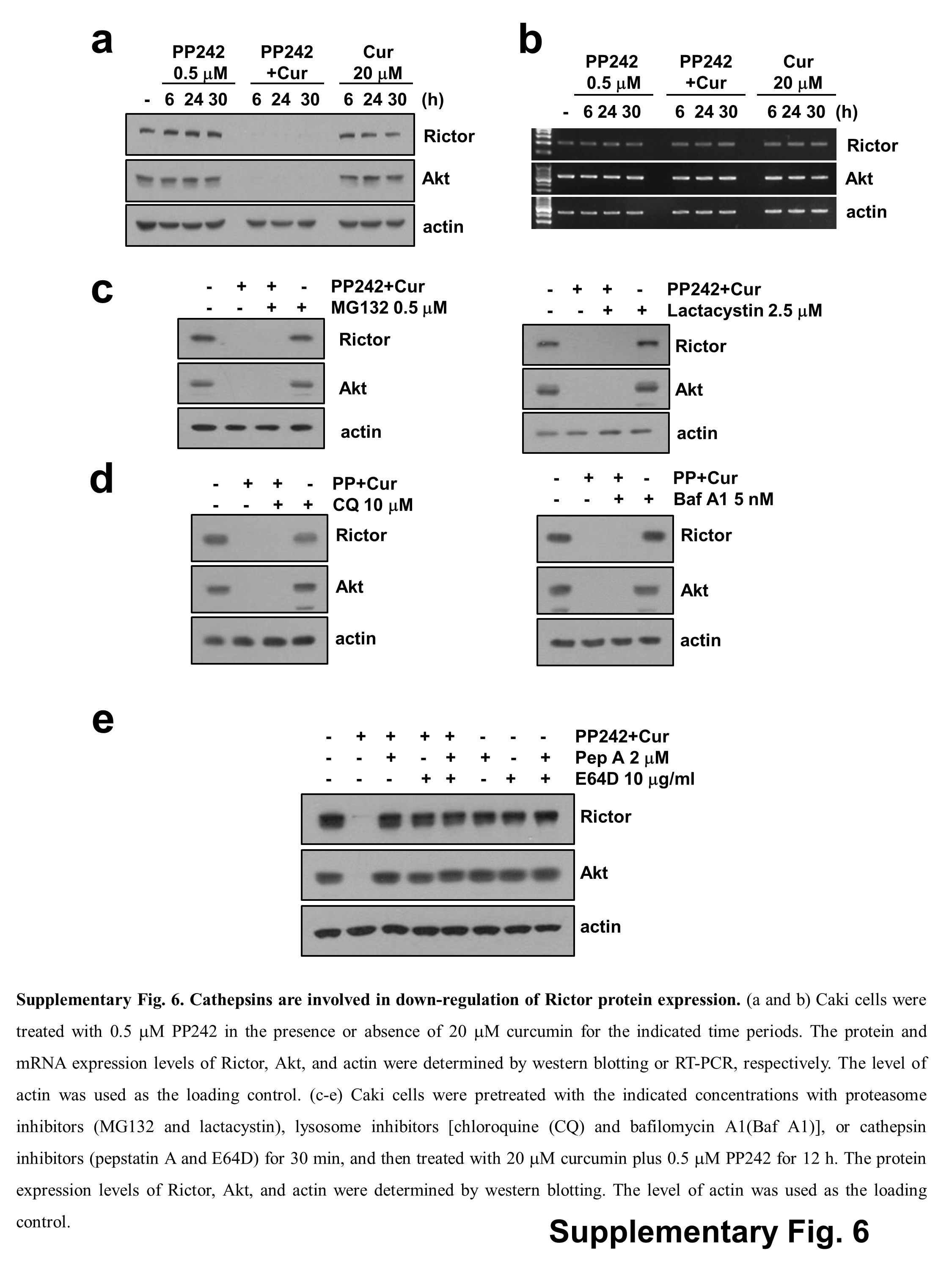

Supplement: Supplementary file 6 — Supplementary Fig.6 [file 41388_2018_345_MOESM6_ESM.tif]

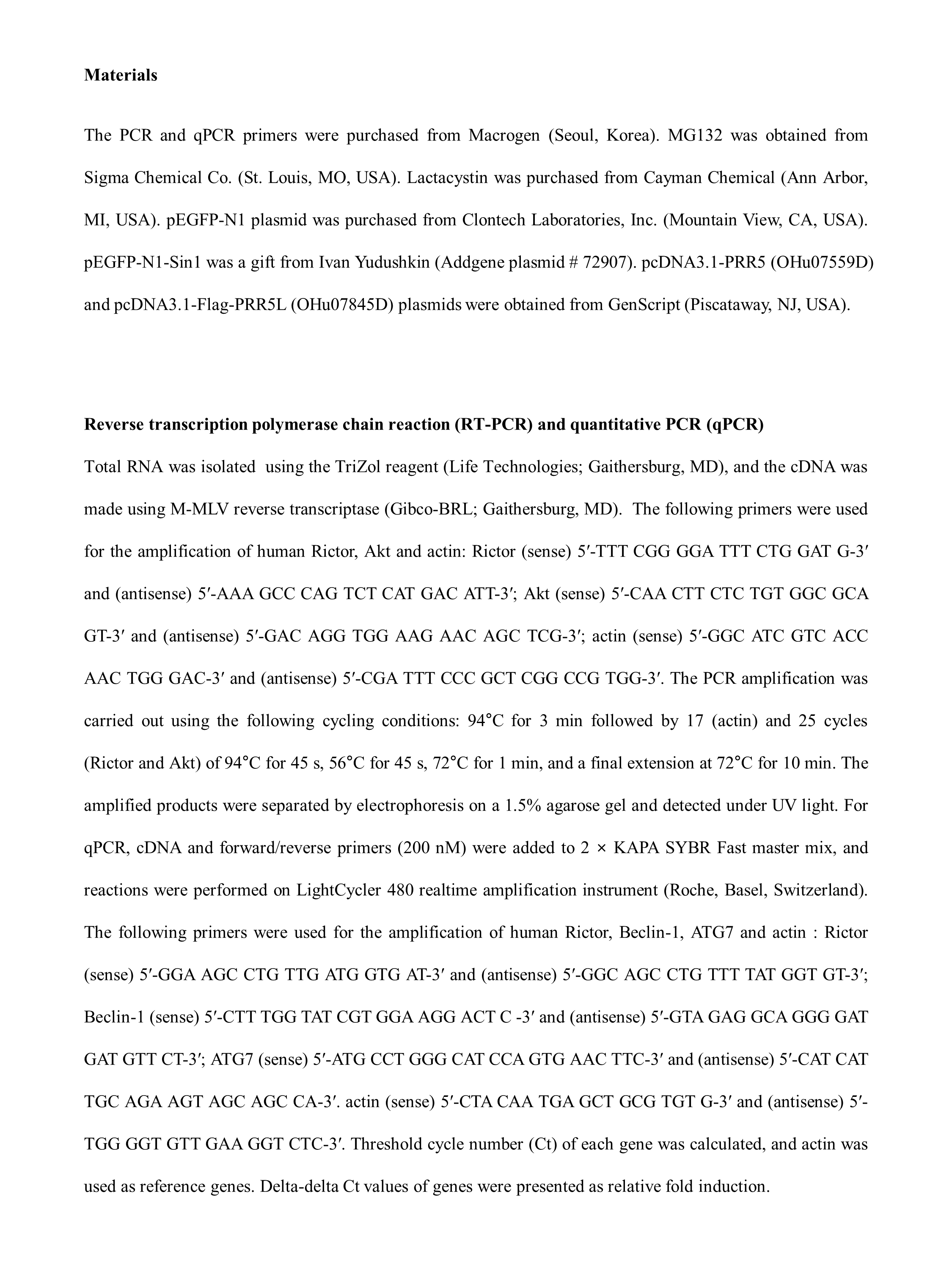

Supplement: Supplementary file 7 — Supplementary infomation [file 41388_2018_345_MOESM7_ESM.tif]

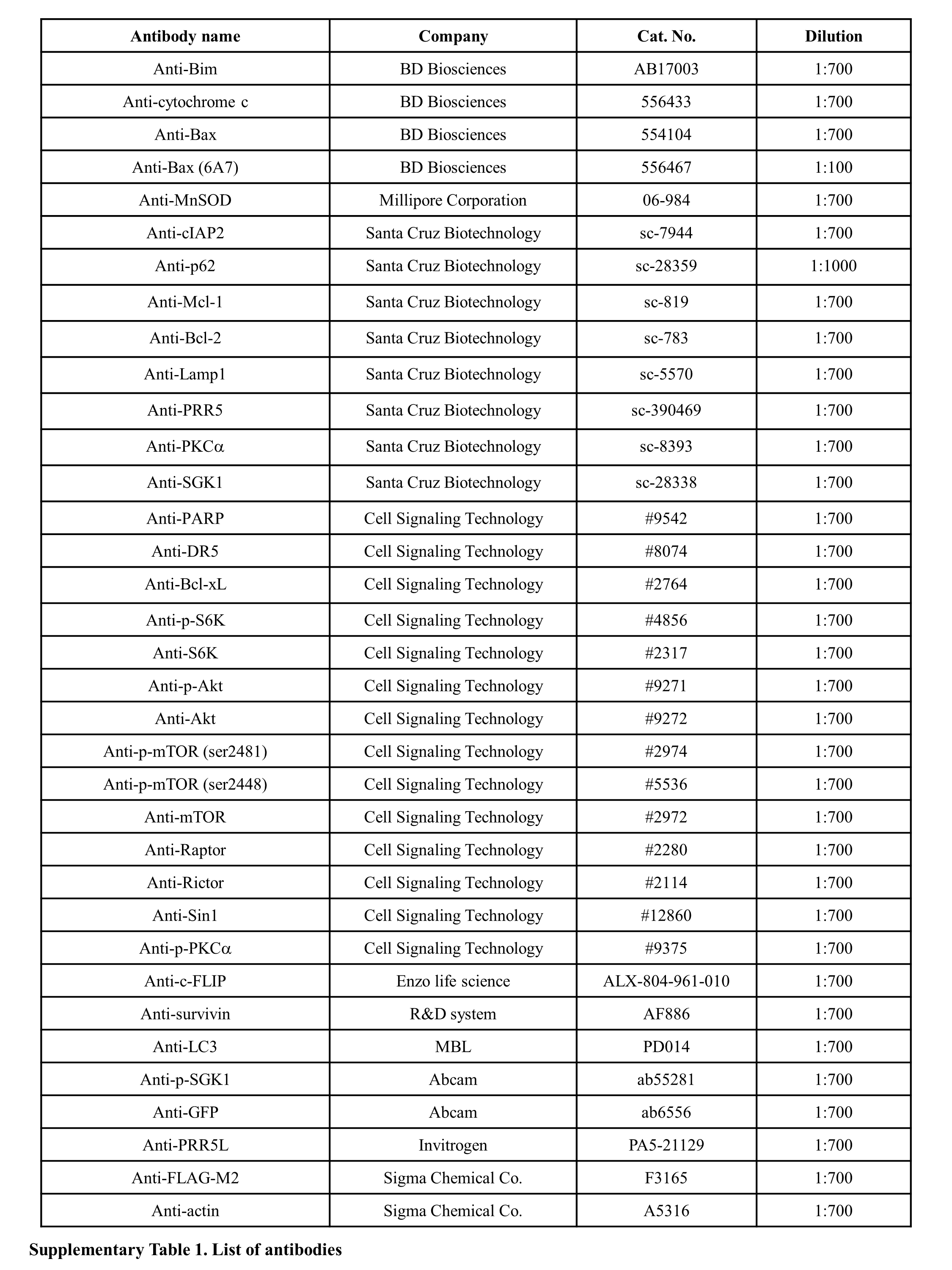

Supplement: Supplementary file 8 — Supplementary table 1 [file 41388_2018_345_MOESM8_ESM.tif]
